# Factors associated with peritoneal metastasis in non-serosa-invasive gastric cancer: a retrospective study of a prospectively-collected database

**DOI:** 10.1186/1471-2407-13-57

**Published:** 2013-02-04

**Authors:** Baojun Huang, Zhe Sun, Zhenning Wang, Chong Lu, Chengzhong Xing, Bo Zhao, Huimian Xu

**Affiliations:** 1 Department of Surgical Oncology, First Hospital of China Medical University, North Nanjing Street 155, Shenyang, 110001, China; 2 Department of Oncological Sciences, Mount Sinai School of Medicine, New York, NY, 10029, USA

**Keywords:** Gastric cancer, Non-serosa-invasive, Peritoneal dissemination, Associated factor

## Abstract

**Background:**

Peritoneal dissemination is the most common type of recurrence in advanced gastric cancer. The main mechanism is thought to be via the exfoliation of free cancer cells (FCCs) from tumor in the gastric serosa. The frequency of recurrence thus increases once the tumor cells penetrate the serosa. However, this type of recurrence also occurs in patients without serosal invasion, though the mechanisms responsible for have not been fully established. We therefore investigated the factors associated with peritoneal dissemination in patients with non-serosa-invasive gastric cancer.

**Methods:**

A total of 685 patients with non-serosa-invasive gastric cancer who underwent curative resection with retrieval of more than 15 nodes were selected. The associations between clinicopathological features and peritoneal dissemination were analyzed. Among them, the tumor infiltrating growth pattern (INF) were classified into α, β and γ according to the Japanese Classification of Gastric Carcinoma (JCGC).

**Results:**

The overall incidence of peritoneal metastasis was 20% (137/685). Age, Borrmann type, differentiation, INF, nodal status and free cancer cells (FCCs) were correlated with peritoneal dissemination using univariate analysis. However, only INF, Borrmann type and TNM node stage were identified as independent correlated factors with peritoneal metastasis by multivariate analysis when FCCs were excluded, and these were also prognostic factors. Peritoneal dissemination was more common in patients with INFγ, Borrmann III/IV and N3 stage. Among patients without FCCs, nodal involvement or vessel invasion, only INF remained an independent associated factor according to multivariate analysis.

**Conclusions:**

Tumor infiltrating growth pattern (INF), together with Borrmann type and TNM node stage, are important factors associated with peritoneal metastasis in non-serosa-invasive gastric cancer.

## Background

Although the incidence of gastric cancer is declining, it remains the second leading cause of cancer-related mortality worldwide. It is also the second most prevalent malignancy in China [1-3]. The incidences of recurrence and metastasis mean that the prognosis for advanced gastric cancer has improved little, despite the use of potentially curative resection. Peritoneal dissemination represents the most common type of recurrence. The main mechanism of peritoneal metastasis is thought to be via exfoliation of free cancer cells (FCCs) from tumor in the gastric serosa, and the frequency of peritoneal metastasis therefore increases once the tumor cells penetrate the serosa, with incidences ranging from 30–60% [4-6]. However, this type of recurrence is also found in patients without serosal invasion. It has been reported that approximately 0.5–2.0% of patients with early gastric carcinoma and 5–11% of patients with non-serosa-invasive gastric carcinoma develop peritoneal recurrence after curative surgery [4,7]. However, the mechanisms responsible for and the factors associated with this type of recurrence remain unknown. Fink and Longmire considered that lymph node dissection opened lymphatic channels and spread viable cancer cells into the intraperitoneal cavity [8]. Kodera et al. thought that cancer cells could infiltrate through the capsule of metastatic lymph nodes to cause metastasis in the peritoneal mesothelium [9]. Tanaka et al. considered that tumor cell obstruction of the lymph vessels could result in the establishment of peritoneal metastasis [10]. All these studies focused on the roles of metastatic lymph nodes in peritoneal dissemination; however, some patients without FCCs, or nodal or vessel involvement still develop peritoneal metastasis. This suggests that some factors other than FCCs and lymph node status may be associated with peritoneal dissemination, though no clear and definite results have yet been reported.

The depth of muscularis propria (MP) invasion is subclassified into superficial MP (sMP) and deep MP (dMP). Patients with dMP invasion have been reported to have similar prognoses to those with subserosal (SS) invasion, while patients with sMP invasion have a better prognosis, similar to early gastric cancer [11]. In the present study, we therefore examined the association between peritoneal dissemination and clinicopathological features in a total of 685 patients with either dMP or SS invasion, to establish the factors associated with peritoneal dissemination.

## Methods

### Patients

This study was a retrospective analysis of a gastric-cancer database collected prospectively from February 1980 to November 2006, at the Department of Surgical Oncology, First Affiliated Hospital, China Medical University. A total of 692 patients with dMP or SS invasion were included, all of whom underwent potentially curative surgery (R0) with the retrieval of more than 15 lymph nodes. At the end of the follow-up period in December 2008, two patients had died during the postoperative period and five patients had been lost to follow-up, giving a follow-up rate of 99%. A total of 685 patients with non-serosa-invasive gastric cancer were therefore enrolled in this study and gave their informed consent. The median follow-up period was 30 months (range 3–297 months).

The follow-up program was performed every 3 months for the first 2 postoperative years, and every 6 months thereafter. The diagnosis of peritoneal recurrence was made on the basis of clinical symptoms, digital examination, or physical, ultrasonographic and radiological findings of bowel obstruction, peritoneal nodules or ascites. All recurrences were confirmed by radiography or histopathology, or both. A total of 137 patients (20%) were diagnosed with peritoneal metastasis during the follow-up period.

### Clinicopathological factors

**Figure 1:**
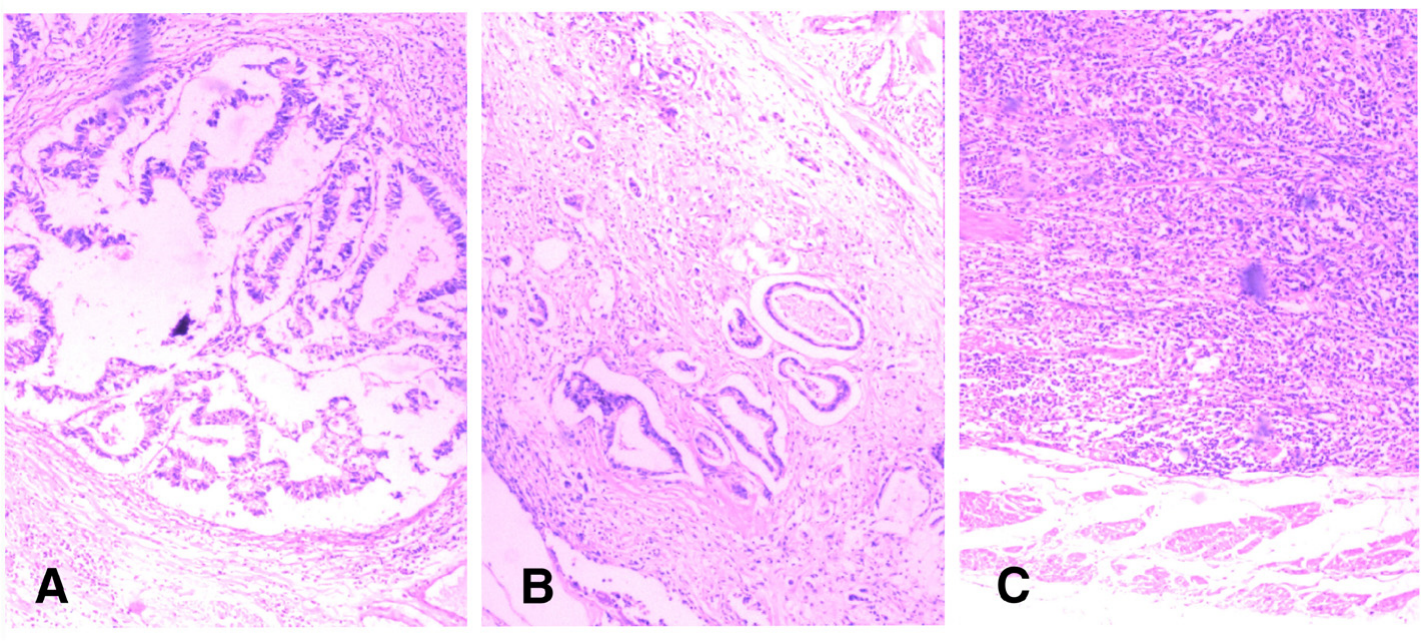
**Predominant pattern of tumor infiltrating growth into the surrounding tissue.** Infiltrating growth pattern was classified as follows: INFα: tumor shows expanding growth and a distinct border with the surrounding tissue (Figure 1**A**); INFγ: tumor shows infiltrating growth and an indistinct border with the surrounding tissue (Figure 1**C**); INFβ: intermediate between INFγ and INFβ (Figure 1**B**) (hematoxylin and eosin, ×40).

The histopathological features were determined by hematoxylin and eosin staining of paraffin sections. Intraoperative cytological examination was performed as follows. Briefly, immediately after the abdominal cavity was opened, 100 ml of physiological saline was introduced into the Douglas cavity, aspirated after gentle stirring, and centrifuged at 2000 rpm for 10 min to collect intact cells. Using the Thin Prep procedure, two slide preparations were made. The first was stained with a modified hematoxylin and eosin preparation and the second using the Papanicolaou method. Each slide was classified as inadequate, negative, or positive. Slide preparations were considered inadequate if there was insufficient cellular material for a diagnosis, and if blood coloration was so heavy that it prevented cellular examination. Sections were reviewed in a double-blind fashion by two highly qualified pathologists to confirm the diagnosis. In the event of a disagreement, the opinion of a third expert was sought who was blinded to both diagnoses. During the study period, 115 samples were eligible for analysis, and a total of 16 patients were confirmed positive for peritoneal FCCs. The cancer cells predominant patterns of infiltrating growth into the surrounding tissue (INF) were classified according to the Japanese Classification of Gastric Carcinoma (JCGC) [12] as follows: INFα: tumor shows expanding growth and a distinct border with the surrounding tissue; INFγ: tumor shows infiltrating growth and an indistinct border with the surrounding tissue; INFβ: intermediate between INFα and INFγ. This classification has been applied in our institute since 1980. Figure 1 shows the detailed pathological characteristics of the three types of INF.

Among the 685 patients, 123 were diagnosed with pathologically-proven dMP invasion and 562 with SS invasion. INFα/β was found in 359 patients (52%), and INFγ in 326 patients (48%). Regarding macroscopic type, Borrmann type I/II was found in 108 patients (16%), and Borrmann type III/IV in 577 patients (84%). The detailed clinicopathological information is listed in Table 1. The study protocol was approved by the Ethics Committee of China Medical University.

**Table 1:** Univariate analysis of peritoneal dissemination in all patients

**Factors**		**Negative (%)**	**Positive (%)**	**χ**^**2**^	***P *value**
Sex	male	394 (82)	88 (18)	3.01	0.08
	female	154 (76)	49 (24)		
Site	lower	359 (80)	91 (20)	0.95	0.81
	middle	74 (83)	15 (17)		
	upper	92 (78)	22 (26)		
	entire	23 (82)	5 (18)		
Age (year)	mean (SD)	58.4 (10.3)	56.7 (12.2)	2.88*	0.09
Size (cm)	mean (SD)	5.4 (2.4)	5.7 (2.5)	1.64*	0.20
Infiltrating pattern	INF α/β	317 (88)	42 (12)	32.49	<0.01
	INF γ	231 (71)	95 (29)		
Differentiation	well/moderate	175 (86)	29 (14)	6.08	0.01
	poor	373 (78)	108 (22)		
Borrmann type	I/II	97 (90)	11 (10)	7.72	<0.01
	III/IV	451 (78)	126 (22)		
Lauren type	intestinal	172 (85)	30 (15)	4.88	0.09
	mixed	17 (81)	4 (19)		
	diffuse	359 (78)	103 (22)		
Depth of invasion	dMP	106 (86)	17 (14)	3.58	0.06
	SS	442 (79)	120 (21)		
Lymphatic/venous invasion	-	444 (80)	109 (20)	0.15	0.69
	+	104 (79)	28 (21)		
TNM stage	N0(0)	170 (86)	27 (14)	23.21	<0.01
	N1 (1–2)	114 (81)	27 (19)		
	N2 (3–6)	128 (85)	23 (15)		
	N3a (7–15)	101 (72)	39 (28)		
	N3b (>15)	35 (63)	21 (37)		
JCGC stage	N0	170 (86)	27 (14)	15.57	<0.01
	N1	173 (83)	36 (17)		
	N2	184 (75)	62 (25)		
	>N3	21 (64)	12 (36)		
FCCs ^#^	negative	84 (85)	15 (15)	27.46	<0.01
	positive	4 (25)	12 (75)		

* Unpaired *t*-test; ^#^ analysis only included 115 patients with intraoperative cytological examination.

### Statistical analysis

Data were analyzed using SPSS statistical software (SPSS, Chicago, IL). Univariate analysis was carried out using the χ^2^ test to determine the significance of differences between categorical variables and unpaired *t*-tests for continuous variables. Multivariate analysis was performed using binary logistic regression with the backward conditional method. Survival analysis was carried out using the Kaplan-Meier estimation and log-rank test. Prognostic factors were assessed using the Cox proportional hazards model. For all analyses, values of *P* < 0.05 were considered significant.

## Results

### Univariate and multivariate analyses of peritoneal metastasis

The incidence of peritoneal metastasis was 14% (17/123) in dMP-invasive and 21% (120/562) in SS-invasive cancers. Among all the patients, age, Borrmann type, differentiation, INF and nodal status (TNM or JCGC node stage) were correlated with peritoneal dissemination according to univariate analysis (Table 1). However, only INF, Borrmann type and TNM node stage were independent associated factors according to multivariate analysis, when FCCs were omitted. The incidence of peritoneal dissemination was much higher in patients with INFγ, Borrmann III/IV, and N3 stage based on UICC/TNM classification. The odd ratios (ORs) of peritoneal metastasis was 2.92 (95% confidence interval [CI]: 1.94–4.40; *P <* 0.001) for patients with INFγ, and 2.06 (95% CI: 1.05–4.04; *P =* 0.035) for patients with Borrmann III/IV, and 2.07 (95% CI: 1.18–3.63; *P* = 0.012) for patients with N3a, and 3.44 (95% CI: 1.71–6.92; *P* = 0.001) for patients with N3b, compared with their counterparts, respectively (Table 2).

**Table 2:** Multivariate analysis of peritoneal metastasis in all patients

**Factors**	**Number of patients**	***P*value**	**OR**	**95.0% CI for OR**	
				**lower**	**upper**
Borrmann type I/II	108		1		
Borrmann type III/IV	577	0.04	2.06	1.05	4.04
Infiltration pattern α/β	359		1		
Infiltration pattern γ	326	<0.01	2.92	1.94	4.40
TNM node stage					
N0 (0)	197		1		
N1 (1–2)	141	0.18	1.50	0.83	2.74
N2 (3–6)	151	0.74	1.11	0.60	2.05
N3a (7–15)	140	0.01	2.07	1.18	3.63
N3b (>15)	56	<0.01	3.44	1.71	6.92

In this database, only 115 patients underwent intraoperative cytological examinations in recent years, and were analyzed further. Among these patients, FCCs in the peritoneal cavity were closely related to peritoneal dissemination according to univariate analysis (Table 1). Moreover, FCCs was the only factor associated with peritoneal metastasis after multivariate analysis (OR=16.80, 95% CI: 4.78-59.10; *P <* 0.001). Borrmann type, INF and TNM node stage were excluded from the model when FCCs was added.

### Multivariate analysis of peritoneal dissemination in patients without FCCs, node metastasis or lymphatic/venous invasion

Regarding lymph node metastasis, lymphatic/venous invasion and FCCs to be important factors influencing peritoneal dissemination, we therefore further assessed the factors associated with peritoneal dissemination in all the patients without nodal involvement, vessel invasion, or positive FCCs. A total of 180 patients were included, 23 of whom had peritoneal dissemination. INF remained an independent correlated factor according to multivariate analysis. The OR of peritoneal metastasis was 3.901 (95% CI: 1.516–10.046; *P* = 0.005) for patients with INFγ compared with INFα or β.

Apart from nodal status and FCCs, INF was the decisive factor associated with peritoneal dissemination in non-serosa-invasive gastric cancer. The relationship between INF and other clinicopathological factors was therefore assessed further. INF was significantly correlated with age, sex, differentiation, Lauren type, TNM node stage and FCCs. INFγ was more common in younger and female patients and those with poor differentiation, diffuse type, N3 stage and positive FCCs (Table 3).

**Table 3:** Relationship between infiltrating growth pattern and other clinicopathological factors

**Factors**		**INFα/β**	**INFγ**	**χ**^**2**^	***P *value**
Sex	male	270 (56)	212 (44)	8.49	<0.01
	female	89 (44)	114 (56)		
Site	lower	240 (53)	210 (47)	4.07	0.25
	middle	40 (45)	49 (55)		
	upper	67 (57)	51 (43)		
	entire	12 (43)	16 (57)		
Age (year)	mean (SD)	59.6 (9.9)	56.4 (11.3)	15.36*	<0.01
Size (cm)	mean (SD)	5.4 (2.2)	5.6 (2.6)	0.62*	0.43
Differentiation	well/moderate	164 (80)	40 (20)	91.21	< 0.01
	poor	195 (41)	286 (59)		
Borrmann type	I/II	65 (60)	43 (40)	3.11	0.08
	III/IV	294 (51)	283 (49)		
Lauren type	intestinal	162 (80)	40 (20)	94.59	< 0.01
	mixed	14 (67)	7 (33)		
	diffuse	183 (40)	279 (60)		
Depth of invasion	dMP	64 (52)	59 (48)	0.01	0.93
	SS	295 (53)	267 (47)		
Lymphatic/venous invasion	-	294 (53)	259 (47)	0.66	0.42
	+	65 (49)	67 (51)		
TNM stage	N0 (0)	110 (56)	87 (44)	9.48	< 0.05
	N1 (1–2)	81 (57)	60 (43)		
	N2 (3–6)	83 (55)	68 (45)		
	N3a (7–15)	59 (42)	81 (58)		
	N3b (>15)	26 (46)	30 (54)		
JCGC stage	N0	110 (56)	87 (44)	4.66	0.20
	N1	116 (56)	93 (44)		
	N2	119 (48)	127 (52)		
	>N3	14 (42)	19 (58)		
FCCs ^#^	negative	55 (56)	44 (44)	7.46	<0.01
	positive	3 (19)	13 (81)		

* Unpaired *t*-test; ^#^ analysis only included 115 patients with intraoperative cytological examination.

### Survival analysis

**Figure 2:**
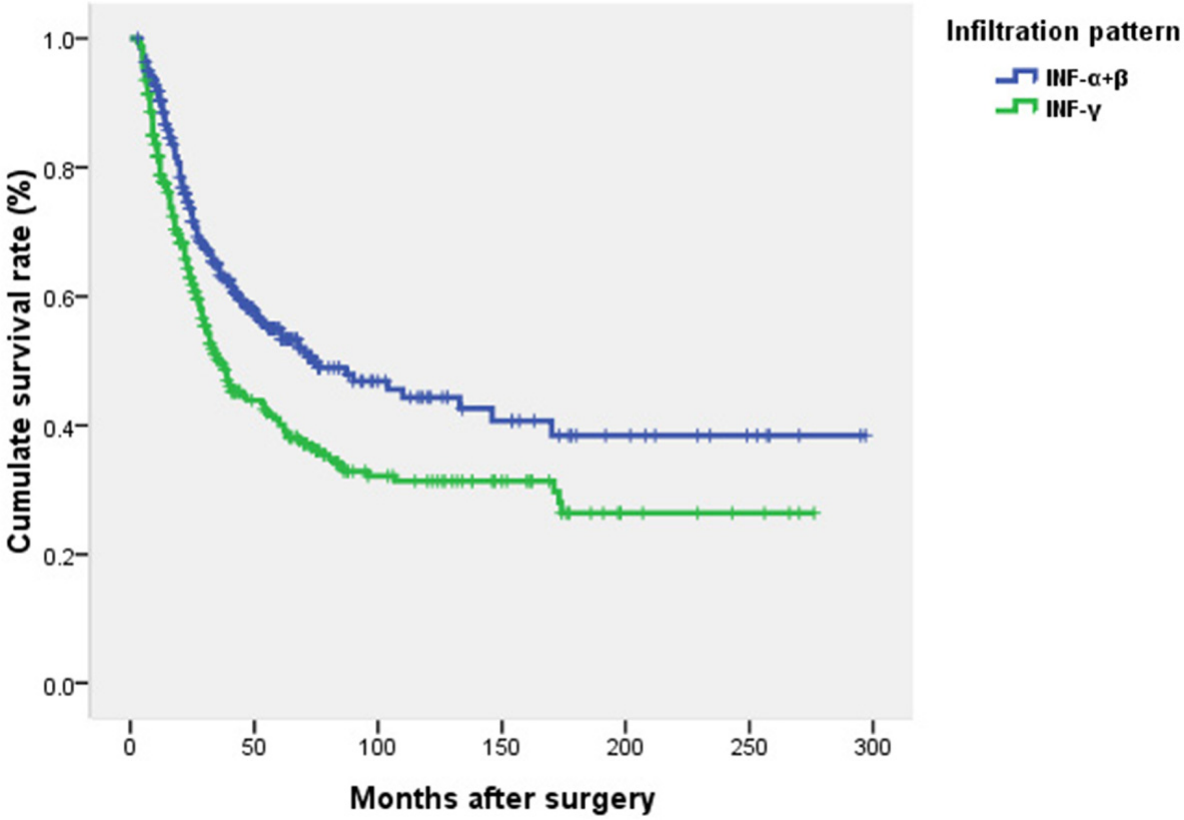
**Survival curves in relation to the tumor infiltrating growth pattern.** Prognosis worsened abruptly in patients with INFγ compared to patients with INFα or β. The difference was significant (χ^2^ = 14.42, *P <* 0.01) using log rank test.

**Figure 3:**
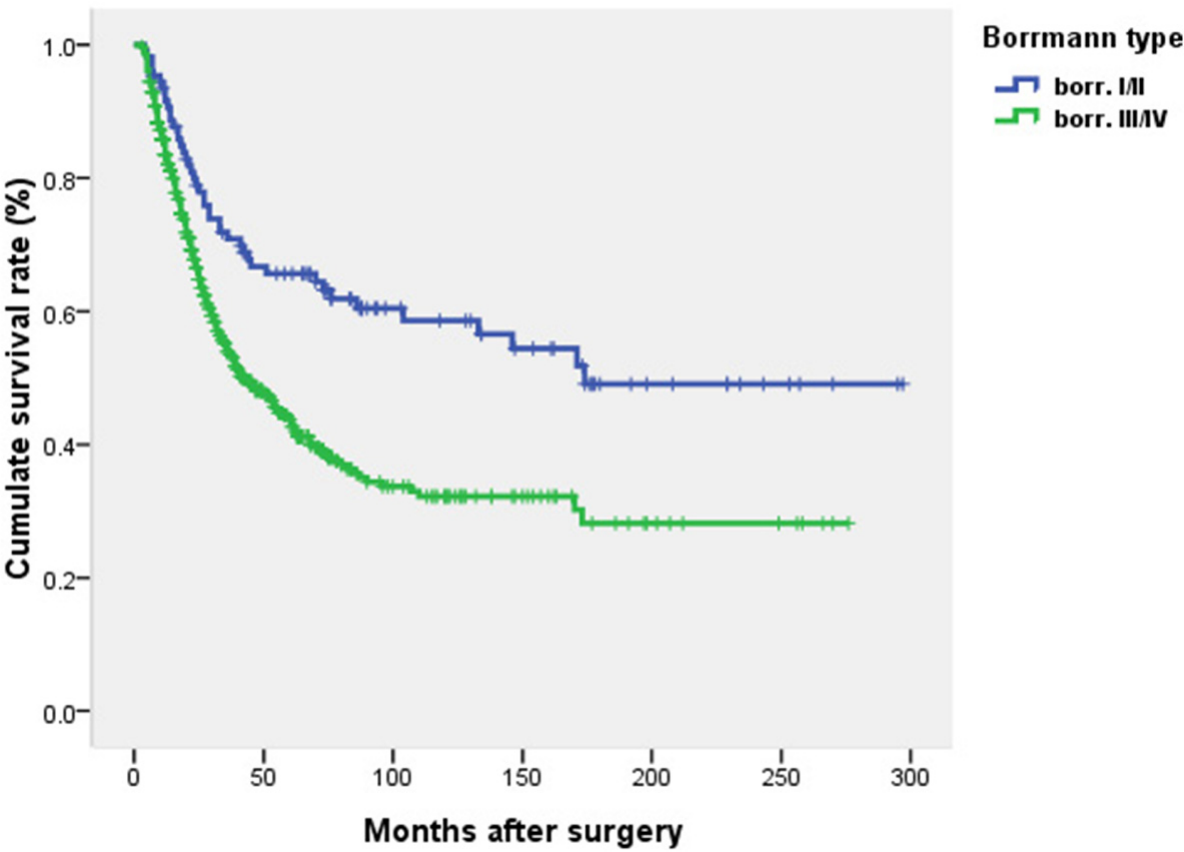
**Survival curves in relation to Borrmann type by Kaplan Meier estimation.** Poorer survival was seen in patients with Borrmann type III/IV compared to those with Borrmann type I/II. The difference was significant (χ^2^ = 16.59, *P <* 0.01) using log rank test.

**Figure 4:**
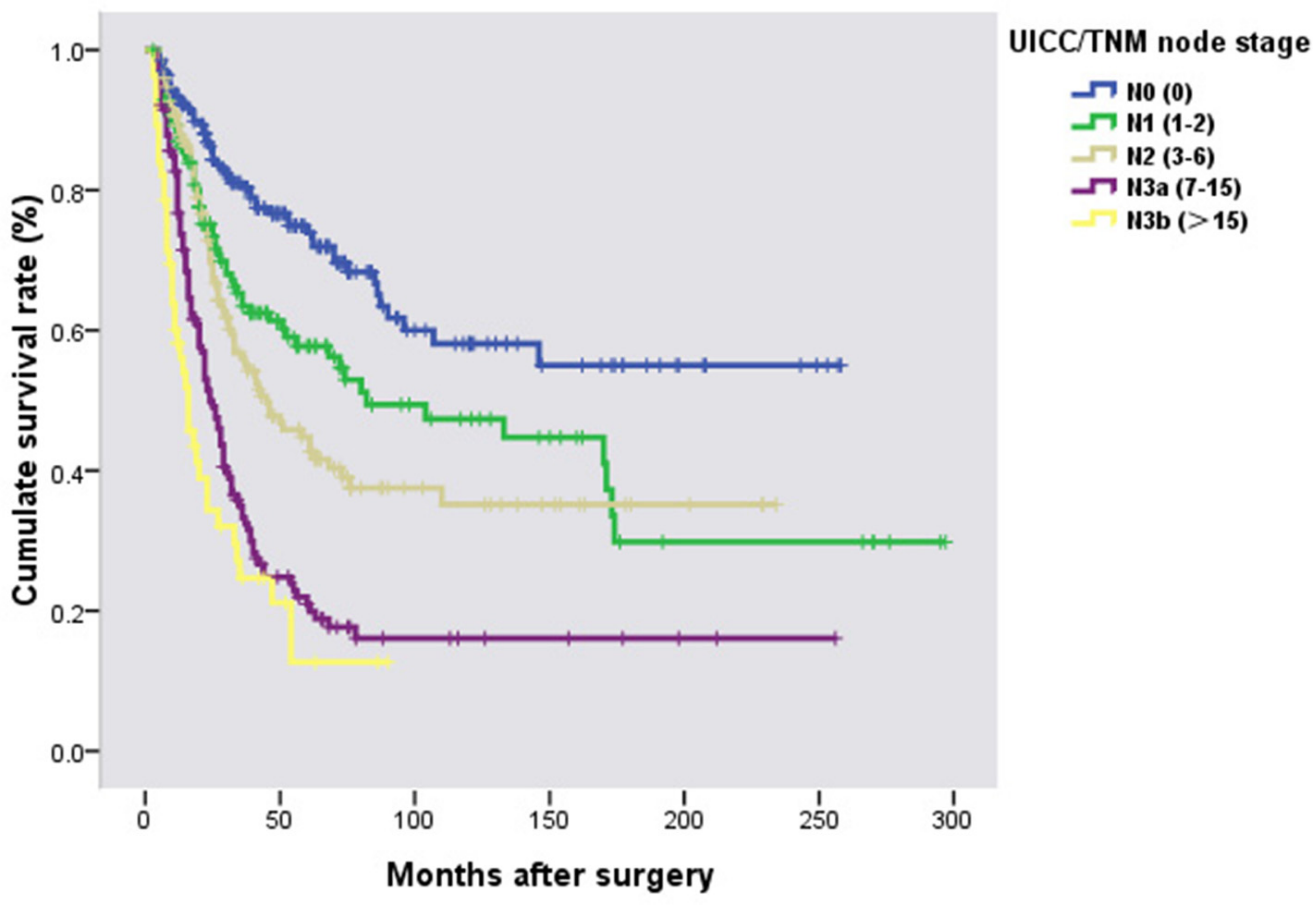
**Survival curves in relation to 7**^**th **^**UICC/TNM node stage.** Prognosis worsened with increasing N stage (χ^2^ = 128.78, *P <* 0.01).

Prognosis was further analyzed among all the patients based on the different clinicopathological factors. When the 12 clinicopathological factors were added into the multivariate Cox regression model simultaneously, INF, Borrmann type, and nodal status were also identified as independent prognostic factors (Table 4). Poorer survival was seen in patients with INFγ (HR 1.41, P < 0.01), Borrmann III/IV (HR 1.60, P < 0.01), and with increased TNM node stage (HR 3.01-6.92, P < 0.01). The survival curves in relation to INF, Borrmann type and TNM node stage are shown in Figures 2, 3, and 4.

**Table 4:** **Multivariate analysis of prognostic factors for disease-free survival in non-serosa-invasive gastric cancer**^**#**^

**Factor**	**Number of patients**	***P*value**	**HR**	**95.0% CI for HR**	
				**lower**	**upper**
Site					
lower	450		1		
middle	89	0.85	1.04	0.73	1.47
upper	118	<0.01	1.47	1.12	1.94
entire	28	0.26	1.32	0.81	2.17
Borrmann type					
I/II	108		1		
III/IV	577	<0.01	1.60	1.16	2.21
Depth of invasion					
dMP	123		1		
SS	562	<0.01	1.57	1.16	2.13
Infiltrating pattern					
INF α/β	359		1		
INF γ	326	<0.01	1.41	1.14	1.75
TNM node stage					
N0 (0)	197		1		
N1 (1–2)	141	<0.01	3.01	1.17	5.31
N2 (3–6)	151	<0.01	3.32	1.97	5.59
N3a (7–15)	140	<0.01	5.54	3.38	9.09
N3b (>15)	56	<0.01	6.92	4.09	11.73

^#^ Determined by multivariate Cox proportional hazards model.

## Discussion

FCCs in the peritoneal cavity is recognized as the most important factor influencing peritoneal dissemination [9,13]. In our clinical practice, cytological examination was performed immediately after the abdominal cavity was opened, which reflected the true state of FCCs in the peritoneal cavity. After surgical dissection, 43°C 4000 ml sterilized distilled water was lavaged in the peritoneal cavity for 10 min. This guaranteed to kill FCCs as much as possible, in order to decrease the incidence of peritoneal dissemination after surgery. Although there was a low incidence of FCCs in non-serosa-invasive gastric cancer, and only 115 patients were analyzed in this study, FCCs were confirmed as the predominant influencing factor for peritoneal metastasis, confirming the direct role of FCCs in the development of peritoneal dissemination. Nodal status is also thought to influence peritoneal metastasis. Theoretically, cancer cells could infiltrate through the capsule of the metastatic nodes or vessel and cause metastasis in the peritoneal mesothelium. Many investigators have reported that lymphatic advancement and lymph node metastasis contribute to the establishment of peritoneal metastasis [9,10,14], and this idea was supported by the results for non-serosa-invasive gastric cancer in the current study. The incidence of peritoneal dissemination increased with increasing TNM node stage. Patients with more than seven metastatic lymph nodes (N3) developed peritoneal dissemination more easily after surgery.

Some factors other than FCCs and nodal status may also be associated with peritoneal dissemination in non-serosa-invasive gastric cancer. The Borrmann classification system was developed in 1926 and is a valuable clinicopathological tool that is widely used to describe endoscopic findings and gross classification. Many studies have shown that Borrmann type correlates with tumor size, depth of invasion, histological grade, lymph node involvement, tumor stage, distant metastasis and prognosis [15-17]. In the present study, patients with Borrmann stage III/IV were more likely to develop peritoneal metastasis than those with Borrmann stage I/II, suggesting increased invasive and metastatic potentials in Borrmann III/IV gastric cancers. Borrmann stage IV gastric cancer has been considered as an independent type in many studies [16,18].

The pattern of infiltrating growth into the surrounding tissue (INF) based on JCGC classification has not been widely used [19]. However, this classification system can be easily utilized after simple training. In the present study, the rate of peritoneal dissemination was higher in patients with INFγ, compared to those with INFα or β. After excluding interference by FCCs, metastatic nodes and vessel invasion, INF remained the only independent factor associated with peritoneal dissemination, thus confirming its role in peritoneal metastasis. A recent study in Korea investigated the role of INF in peritoneal metastasis in pT2b patients and confirmed that infiltrative-type growth pattern was closely related to peritoneal dissemination [20]. INF thus represents a promising predictive factor for peritoneal metastasis in non-serosa-invasive gastric cancer.

The reasons why INF plays such an important role in peritoneal metastasis in non-serosa-invasive gastric cancer remains unclear. In the present study, INF was positively correlated with FCCs, Lauren type, differentiation and TNM node stage. INFγ was more common in patients with FCCs, diffuse type, poor differentiation and late node stage (N3), all of which reflect malignant behavior. Thus INFγ tumors were more invasive than INFα or β tumors, which could partly account for this phenomenon, based on clinicopathological study. However, further molecular biological studies are needed to confirm the precise mechanisms involved.

In this study, prognosis was assessed based on clinicopathological factors, and INF, Borrmann type, and nodal status were determined to be independent prognostic factors. The results of this study identified these three factors as independent predictors for both prognosis and peritoneal recurrence, thus confirming their valuable roles in clinical practice.

## Conclusions

The results of this study suggest that tumor infiltrating growth pattern (INF), together with Borrmann type and UICC/TNM node stage, are important factors associated with peritoneal metastasis in non-serosa-invasive gastric cancer. Patients with INFγ, Borrmann stage III/IV, and N3 stage should be closely followed-up to detect peritoneal dissemination.

## Abbreviations

INF: Tumor infiltrating growth pattern; FCCs: Free cancer cells; MP: Muscularis propria; sMP: Superficial MP; dMP: Deep MP; SS: Subserosal invasion; HRs: Hazard ratios; ORs: 0dd ratios.

## Competing interests

The authors declare that they have no competing financial or other interests.

## Authors' contributions

BH and HX conceived the study, analyzed data, and drafted the manuscript and submitted the manuscript. ZS, ZW, CL and CX revised the manuscript critically for important intellectual content. BZ conceived the study and helped to draft the manuscript. All authors read and approved the final manuscript.

## Pre-publication history

The pre-publication history for this paper can be accessed here:

http://www.biomedcentral.com/1471-2407/13/57/prepub
